# Diagnostic Value of Correlation Between Canine Chronic Enteropathy Clinical Activity Index (CCECAI) and Selected Hematological and Immunological Biomarkers in Dogs with Chronic Enteropathy: A Systematic Meta-Analysis

**DOI:** 10.3390/vetsci13040316

**Published:** 2026-03-26

**Authors:** Mohamed Marzok, Ghada Ashraf, Adel Almubarak, Hussein Babiker, Sabry El-khodery

**Affiliations:** 1 Department of Clinical Sciences, College of Veterinary Medicine, King Faisal University, Al-Ahsa 31982, Saudi Arabia; aalmubarak@kfu.edu.sa (A.A.); hbabiker@kfu.edu.sa (H.B.); 2 Department of Internal Medicine and Infectious Diseases, School of Veterinary Medicine, Badr University in Cairo (BUC), Badr City 32879, Egypt; ghadaashraf.ga@gmail.com; 3 Department of Internal Medicine and Infectious Diseases, Faculty of Veterinary Medicine, Mansoura University, Mansoura 35516, Egypt

**Keywords:** biomarkers, canine, CIE, hematology, IBD

## Abstract

C-reactive protein (CRP) level, platelet-to-lymphocyte ratio (PLR), and neutrophil-to-lymphocyte ratio (NLR) have been proposed as biomarkers that reflect disease severity in canine chronic enteropathies. Their correlation with the Canine Chronic Enteropathy Clinical Activity Index (CCECAI) remains unclear. The objective of the present meta-analysis was to assess the correlation between CCECAI and hematological and immunological markers in dogs with CIE. A meta-analysis was conducted to evaluate the correlation of CRP, PLR, and NLR with CCECAI as a diagnostic marker. The PRISMA guidelines were followed to accomplish this procedure. Pooled correlation coefficients were calculated using Fisher’s Z-transformation under random effects models. Heterogeneity and publication bias were examined using standard procedures for the meta-analysis. Eleven studies were found to be fit: five for CRP, four for NLR, and four for PLR. The pooled correlation for CRP level was 0.229 (95% CI: 0.143–0.311, *p* < 0.001), with consistent findings across studies (I^2^ = 0%). PLR demonstrated a moderate correlation (pooled r = 0.381, 95% CI: 0.238–0.508, *p* < 0.001; I^2^ = 41%), while NLR showed a slightly stronger correlation (pooled r = 0.410, 95% CI: 0.286–0.521, *p* < 0.001) but with substantial heterogeneity (I^2^ = 68%). Publication bias analyses indicated modest asymmetry for CRP and PLR, with Egger’s regression significant for both, whereas the NLR showed no clear evidence of bias. Trim-and-fill adjustments slightly attenuated the pooled estimates but did not alter the statistical significance. In conclusion, the correlation between CCECAI and NLR is the most promising biomarker, followed closely by PLR, whereas CRP has a weaker predictive value. The correlation between CCECAI and each of NLR and PLR may provide rapid and reliable diagnostic information about CIE in dogs.

## Simple Summary

The purpose of the present meta-analysis was to assess the correlation of C-reactive protein (CRP) level, platelet-to-lymphocyte ratio (PLR), and neutrophil-to-lymphocyte ratio (NLR) with the Canine Chronic Enteropathy Clinical Activity Index (CCECAI) as diagnostic markers in dogs with chronic inflammatory enteropathy (CIE). Following the PRISMA guidelines, 11 studies were included for evaluation (CRP level, 5; PLR, 4; NLR, 4). Pooled correlations under random-effects models showed a weak correlation with CRP (r = 0.229), a moderate correlation for PLR (r = 0.381), and a strong correlation for NLR (r = 0.410). There was no publication bias for NLR. However, publication bias was modest for CRP and PLR but did not affect significance. Overall, NLR and PLR demonstrated greater correlation than CRP. The results of the present meta-analysis indicate that the correlation between CCECAI and each of NLR and PLR may provide rapid and reliable diagnostic information about CIE in dogs.

## 1. Introduction

Chronic inflammatory enteropathies (CIEs) are a collection of diseases with an indefinite origin that are thought to evolve from a combination of aberrant immune responses and disruptions in microbiota–host interactions in genetically liable individuals [1]. The disease can be categorized into immunosuppressant microbiota-modulation-responsive and food-responsive diseases [2]. Protein-losing enteropathies (PLEs) are the terms used when CIE is associated with low serum albumin levels. According to their response to therapy, PLEs can be subcategorized into food-responsive PLEs, immunosuppressant-responsive PLEs, or non-responsive PLEs [3,4]. Regarding treatment of CIE, there is no definitive therapy for this disease condition, and the intervention focuses mainly on alleviating clinical signs [5].

Diagnosis of CIE is based on clinical examination with the establishment of the Canine Chronic Enteropathy Clinical Activity Index (CCECAI) [6,7], clinical pathology [8,9], and endoscopic examination with subsequent histopathological characterization [10,11,12,13].

CCECAI is a standardized scoring system used in veterinary medicine to evaluate the severity of chronic gastrointestinal disorders in dogs, providing a structured way to monitor clinical signs, assess treatment response, and ensure consistency in research reporting [2,7]. It integrates clinical parameters such as activity level, appetite, vomiting, stool characteristics, weight loss, serum albumin, and presence of ascites or edema, each scored on a numerical scale [1,6].

CCECAI is not an adequate diagnostic marker because it is subjective and variable [14], does not diagnose etiology [15], correlates poorly with histopathology [16], underrepresents biochemical markers [17], and lacks standardization across studies [18]. Although confirmation of CIE depends mainly on histopathological examination [19,20], histopathology results vary according to the stage of the disease [21]. An endoscopic biopsy should be performed to conduct histopathological examinations. Although this step can provide a robust diagnostic tool, it is expensive, time-consuming, and may pose risks [14].

Alternatively, in association with CCECAI, hematological and immunological parameters have been measured to improve the diagnostic tools of CIE. Immunological and hematological examinations include C-reactive protein [22], fecal calprotectin [6,23,24], neutrophil-to-lymphocyte ratio (NLR) [25], platelet-to-lymphocyte ratio (PLR) [26], and albumin [27]. However, such parameters have been found to provide variable correlations with CCECAI. To assess the feasibility of using the correlation between CCECAI and hematological and immunological parameters as a rapid, reliable, and safe diagnostic aid for CIE, robust conclusions should be obtained.

Meta-analysis is a statistical procedure that links results from various independent studies to produce a single, more powerful conclusion regarding a research question. It offers a powerful technique to assess the diagnostic significance of hematological and immunological parameters in canine patients, as it increases statistical power, resolves variations across studies, and provides evidence-based conclusions that can guide veterinary diagnostics and research [28,29,30]. To the best of our knowledge, there is no available meta-analytical study on this topic. Therefore, the main objective of the present meta-analysis was to assess the correlation between CCECAI and hematological and immunological markers in dogs with CIE.

## 2. Materials and Methods

### 2.1. Guideline

This study was conducted according to the Preferred Reporting Items for Systematic Reviews and Meta-Analyses guidelines [31]. The PRISMA Checklist was followed for each section of the meta-analysis (https://www.prisma-statement.org/).

### 2.2. Animals

The present investigation incorporated all articles that focused on the correlation between the CCECAI and CRP, NLR, and PLR. The dogs enrolled in this study had chronic enteropathy. Confirmation of chronic enteropathy was based on histopathological findings.

### 2.3. Search Strategy

As per the standards of Preferred Reporting Items for Systematic Review and Meta-analyses (PRISMA), we conducted a systematic review and meta-analysis to obtain all the published literature, including preprints and gray literature. We searched all four major databases (Scopus, PubMed, Web of Science, and Google Scholar) using the following keywords: “C-reactive protein” OR “CRP” OR “Neutrophil to lymphocyte ratio” OR “NLR” OR” Platelet to lymphocyte ratio” OR “PLR” and “Canine Chronic Enteropathy Clinical Activity Index” OR “CCECAI” The last update of the search was performed on 27 January 2026. There were no restrictions on language or year of publication in the search strategy.

### 2.4. Inclusion Criteria

▪ Studies included clinical examination with the establishment of CCECAI, clinicopathological findings, and endoscopic examination with surgical collection of GI tissue biopsies.
▪ Studies estimated the levels of CRP level, NLR, and PLR and correlated them with CCECAI in dogs with chronic enteropathy.
▪ Case–control studies.
▪ Studies included diagnosis of chronic enteropathy according to the guidelines of the World Small Animal Veterinary Association [32].

*Exclusion criteria*

▪ Studies reported validation of the reagents and instruments.
▪ Studies on pets other than dogs.
▪ Studies with insufficient data.
▪ Reviews, case reports, and letters to the editor were excluded.

### 2.5. Data Extraction and Quality Assurance

Study screening was conducted using the EndNote software version 9 [33]. First, duplicates were excluded. The titles and abstracts of the papers discovered during the initial database search were independently examined by two authors who focused on those that were closely linked.

The full texts of the studies were given to and assessed by the same authors. The meta-analysis included studies that met these requirements. To examine whether there was any further pertinent research, we searched the references of relevant publications. A third party was brought in to resolve conflicts between the two screeners. Two reviewers independently gathered the following data from the included articles: study design and location, initial author name, publication year, and number of cases. The level of diseased cases, or sufficient information to estimate the correlation, was used by two authors to independently evaluate the quality of the listed studies [34].

### 2.6. Statistical Analysis

Statistical analysis was carried out using specified software (Comprehensive Meta-Analysis ver. 2, National Institute of Health, Bethesda, MD, USA). The pooled effect size for the correlation between CCECAI and hematological and immunological parameters was identified by processing the correlation and Fisher’s Z test. The random effects model was used in this investigation as there was no heterogeneous outcome among studies. To assess heterogeneity, we used both the Q and I^2^ statistics. Results were indicative of significant heterogeneity if their I^2^ value was greater than 75% and their *p*-value was <0.05. Publication bias was identified by processing the funnel plot with standard error and precision. Additionally, Egger’s linear regression test, Begg and Mazumdar rank correlation, and Fail-safe N test were used to confirm evidence of publication bias. *p*-value less than 0.05 was considered indicative of statistical significance.

## 3. Results

### 3.1. Pooled Correlation

**Figure 1:**
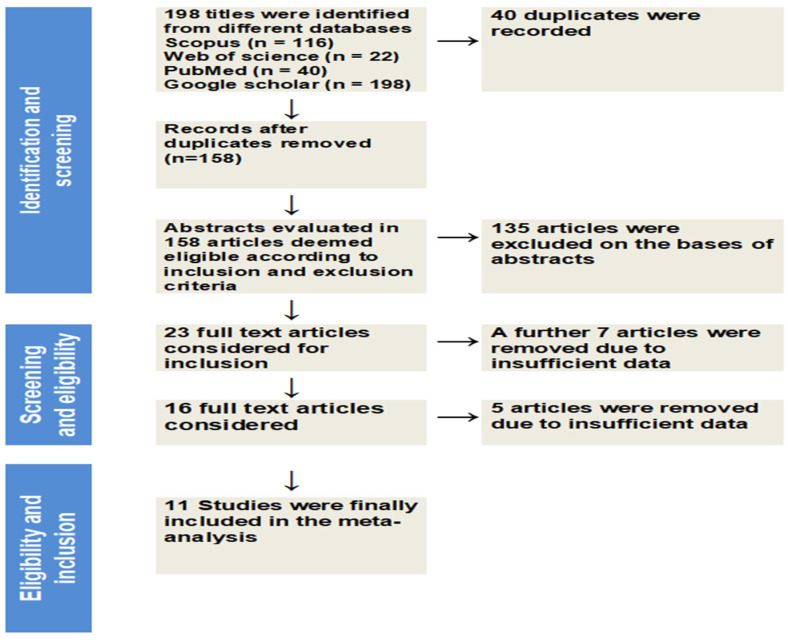
PRISMA flow diagram of study selection regarding the correlation between CCECAI and CRP, NLR, and PLR in dogs with enteropathy. From 198 records identified across databases, 158 abstracts were screened, 23 full texts assessed, and 11 studies were included in the final meta-analysis.

The search results of the selected databases yielded 198 items. Eleven studies, with 780 dogs, were finally included in the meta-analysis after duplicates and irrelevant records were eliminated. The PRISMA flow diagram, shown in Figure 1, provides details of the inclusion and exclusion procedures.

For the correlation between CCECAI and CRP, five studies met the selection criteria (Table 1). For the correlation between CCECAI and NLR, four studies were selected (Table 2). However, for the correlation between CCECAI, NLR, and PLR, only four studies for each item were finally selected (Table 3).

**Figure 2:**
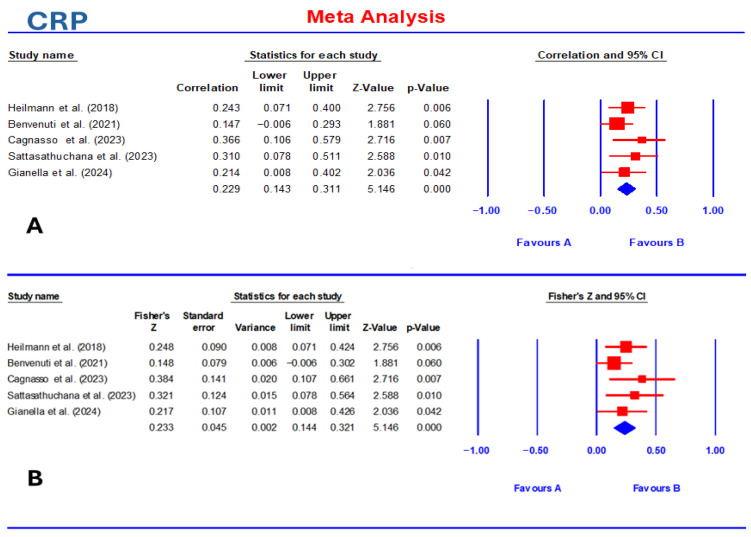
Forest plot summarizing pooled effect sizes and heterogeneity for the correlation between CCECAI and CRP (**A**) [6,7,8,22,35] and Fisher’s Z value (**B**) [6,7,8,22,35] in dogs with chronic enteropathy. The biomarker was displayed with its random effects estimate and 95% confidence interval. CRP yielded a smaller pooled effect (0.23, 95% CI: 0.14–0.31) and no heterogeneity (I^2^ = 0).

Five studies were included in the meta-analysis to evaluate the correlation between CRP and CCECAI. The individual study correlations ranged from 0.147 to 0.366. Four studies (Heilmann et al. [6]; Cagnasso et al. [8]; Sattasathuchana et al. [35]; Gianella et al. [22]) demonstrated statistically significant positive associations, whereas one study (Benvenuti et al. [7]) reported a non-significant correlation (Table 1). The pooled correlation coefficient was 0.229 (95% CI: 0.143–0.311; Z = 5.146, *p* < 0.001). Fisher’s Z transformation yielded a comparable pooled estimate (Z = 0.233, 95% CI = 0.144–0.321), confirming the consistency of the findings (Figure 2).

**Figure 3:**
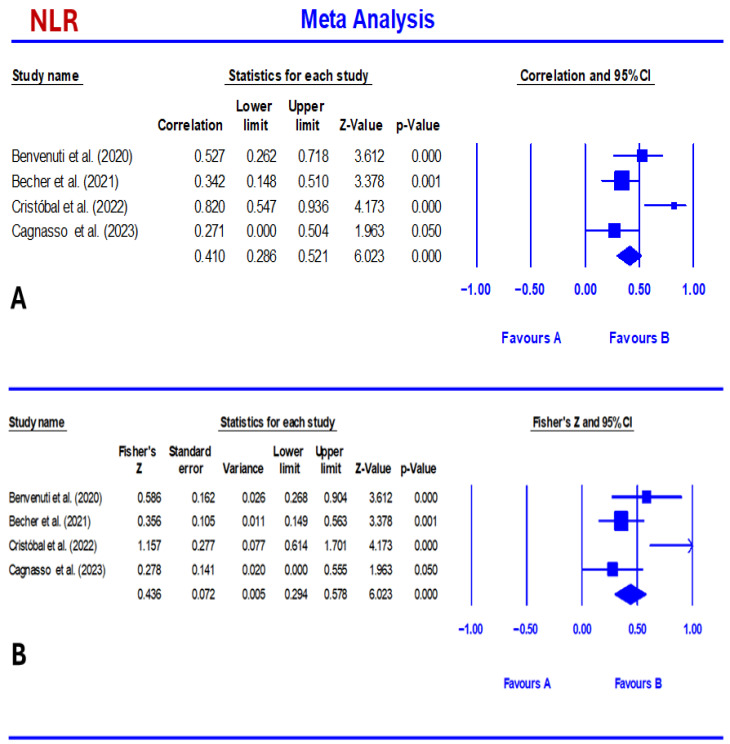
Forest plot summarizing pooled effect sizes and heterogeneity for the correlation between CCECAI and NLR (**A**) [7,27,36,39] and Fisher’s Z value (**B**) [7,27,36,39] in dogs with chronic enteropathy. The biomarker was displayed with its random effects estimate and 95% confidence interval. NLR demonstrated a significant pooled effect (0.47, 95% CI: 0.24–0.66) with substantial heterogeneity (I^2^ = 68%).

**Figure 4:**
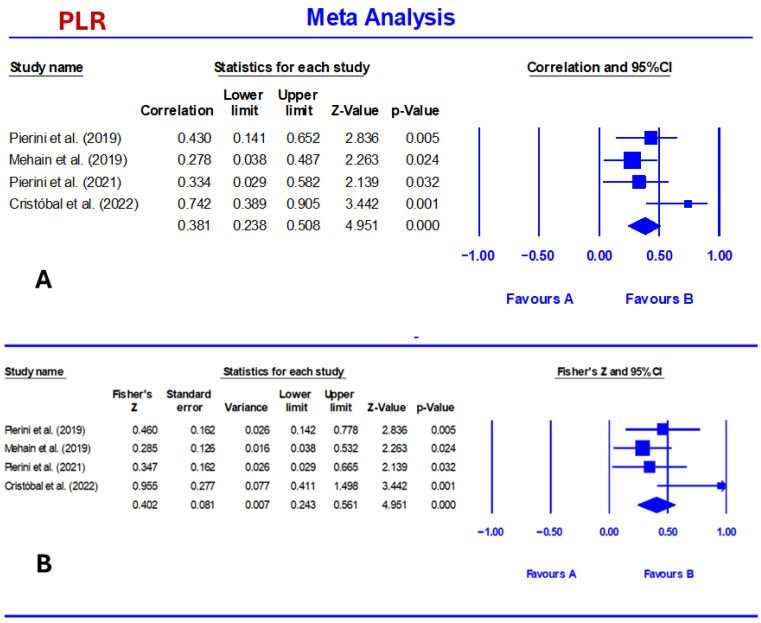
Forest plot summarizing pooled effect sizes and heterogeneity for the correlation between CCECAI and PLR (**A**) [26,36,37,38] and Fisher’s Z value (**B**) [26,36,37,38] in dogs with chronic enteropathy. The biomarker was displayed with its random effects estimate and 95% confidence interval. PLR demonstrated a significant pooled effect (0.23, 95% CI: 0.14–0.31) and no heterogeneity (I^2^ = 0%).

NLR showed a moderate to strong association, which was slightly stronger than that of PLR. At the same time, the pooled correlation between CCECAI and NLR was 0.410 (95% CI: 0.286–0.521, *p* < 0.001). Correlations ranged from 0.271 to 0.820 (Table 2; Figure 3). Fisher’s Z pooled estimate was 0.436 (95% CI: 0.294–0.578, *p* < 0.001). PLR showed a moderate and statistically significant association with CCECAI. Thus, the pooled correlation was 0.381 (95% confidence interval [CI]: 0.238–0.508, *p* < 0.001). The pooled correlation coefficient ranged from 0.278 to 0.742 (Table 3 and Figure 4). The Fisher’s Z pooled estimate was 0.402 (95% CI: 0.243–0.561, *p* < 0.001).

### 3.2. Heterogeneity

Across biomarkers, for NLR, the random-effects estimate was 0.41 (95% CI: 0.24–0.66, *p* < 0.001), but heterogeneity was substantial (I^2^ = 68%, Q = 9.45, *p* = 0.024), indicating considerable variability across studies. PLR showed a similar magnitude of effect (0.43, 95% CI: 0.22–0.65, *p* < 0.001) with moderate heterogenicity (I^2^ = 41%, Q = 5.08, *p* = 0.166), suggesting greater consistency than NLR. In contrast, CRP yielded a smaller pooled effect (0.229, 95% CI: 0.14–0.31, *p* < 0.001), but with no heterogeneity (I^2^ = 0%, Q = 2.87, *p* = 0.58), reflecting highly consistent findings across studies (Table 4).

### 3.3. Publication Bias

**Figure 5:**
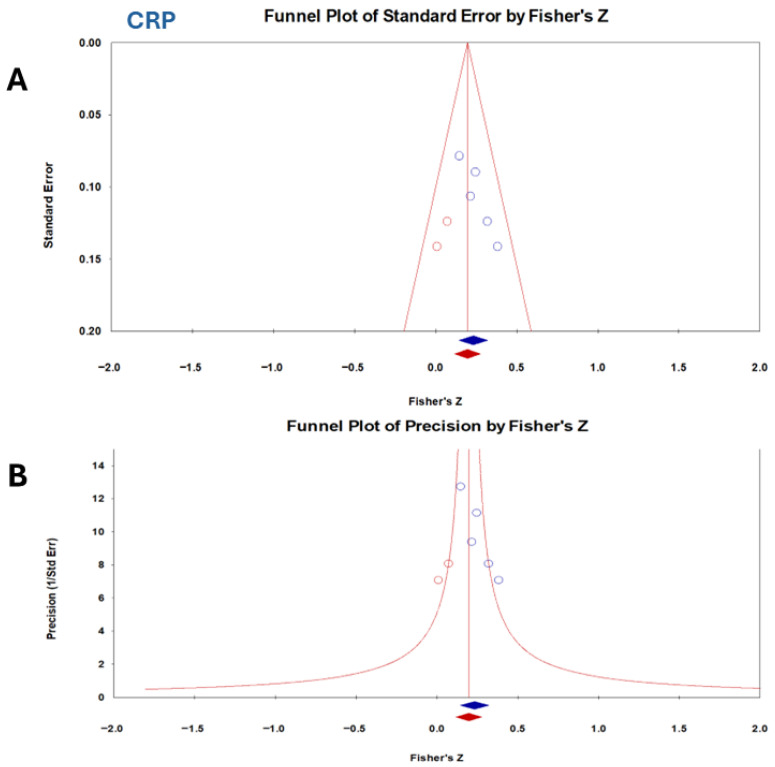
Funnel plot assessing publication bias for the correlation between CCECAI and CRP in dogs with chronic enteropathy. Each point represents an individual study plotted against its effect size with standard error (**A**) or precision (**B**). Visual inspection shows relative symmetry for CRP, consistent with the absence of heterogeneity (I^2^ = 0%).

Publication bias was evaluated using several methods. For CRP, Begg and Mazumdar’s rank correlation test revealed a significant funnel plot asymmetry (Kendall’s S = 8.00, τ = 0.80, corrected τ = 0.70), indicating that smaller studies tended to report larger effect sizes (Figure 5). The Egger’s regression test confirmed this asymmetry (intercept = 3.36, *p* = 0.028). The Duval and Tweedie trim-and-fill method imputed several potentially missing studies, with the pooled effect size decreasing only slightly from 0.22 to 0.19.

**Figure 6:**
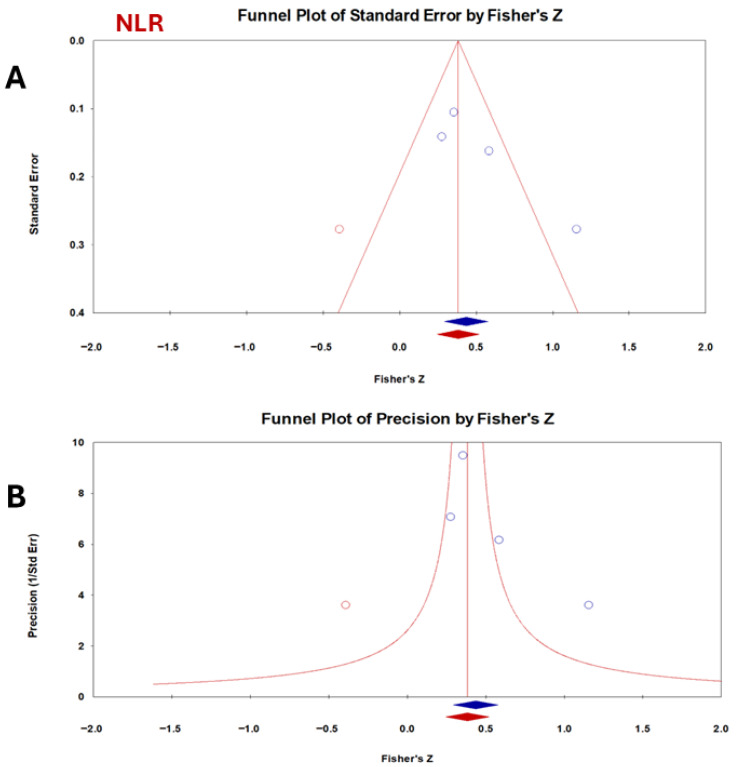
Funnel plot assessing publication bias for the correlation between CCECAI and NLR in dogs with chronic enteropathy. Each point represents an individual study plotted against its effect size with standard error (**A**) or precision (**B**). NLR shows greater asymmetry and variability (I^2^ = 68%), suggesting possible small-study effects.

For NLR, Egger’s regression test showed a positive intercept (4.4), although it was not statistically significant (*p* = 0.11), suggesting no clear evidence of funnel plot asymmetry (Figure 6). The Classic fail-safe N analysis estimated that 41 additional null studies would be required to render the observed effect nonsignificant, supporting the robustness of the findings despite the limited number of included studies (n = 4). Duval and Tweedie’s trim-and-fill procedure produced a slightly attenuated pooled effect size (observed = 0.40, adjusted = 0.36).

**Figure 7:**
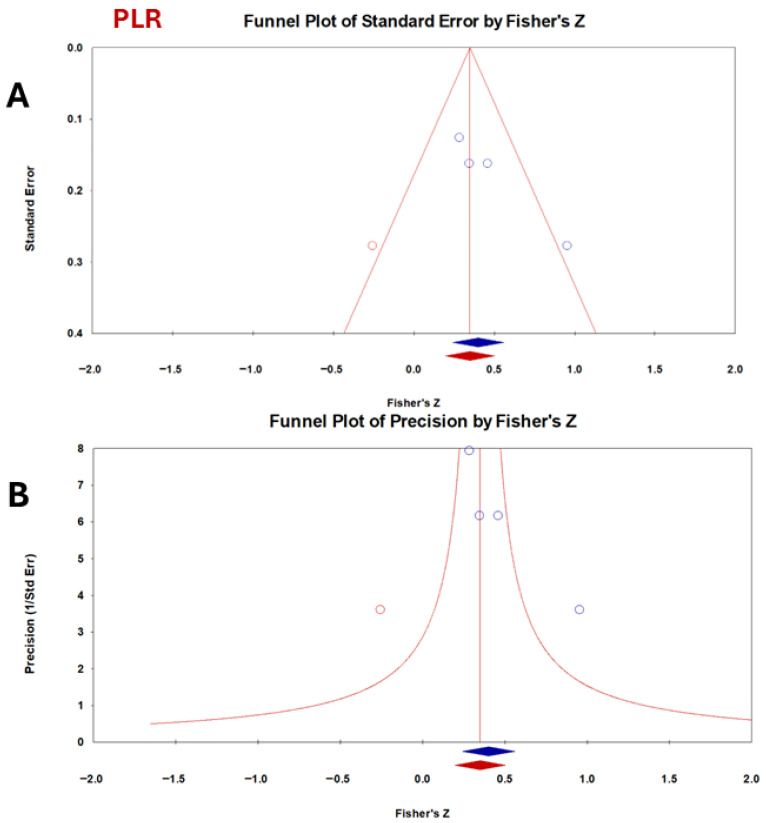
Funnel plot assessing publication bias for the correlation between CCECAI and PLR in dogs with chronic enteropathy. Each point represents an individual study plotted against its effect size with standard error (**A**) or precision (**B**). PLR demonstrates moderate dispersion (I^2^ = 41%).

For PLR, Begg and Mazumdar’s rank correlation test showed a positive tau coefficient but did not reach statistical significance, suggesting possible small-study effects without conclusive evidence. In contrast, Egger’s regression intercept was statistically significant, indicating funnel plot asymmetry and raising concerns for bias among smaller studies (Figure 7). Fail-safe N analysis demonstrated that 26 additional null studies would be required to overturn the observed significance, whereas Orwin’s test confirmed that the mean effect size remained above a trivial threshold.

## 4. Discussion

In veterinary medicine, the term chronic enteropathy (CE) is used to describe dogs with persistent gastrointestinal signs lasting longer than three weeks, after the exclusion of other causes. CE encompasses several subtypes based on therapeutic responses, including food-, antibiotic-, and immunosuppressant-responsive enteropathies [7]. Food-responsive enteropathy is the most common CIE treatment subtype, followed by immunosuppressive-responsive enteropathy (IRE, 30%) and non-responsive enteropathy [40].

Effective diagnostic and prognostic decisions for canine CE depend mainly on the selection of correct, rapid, and accurate diagnostic tools [41]. As CCECAI has found insufficient diagnostic markers, clinicopathological markers were added to obtain rapid decisions. Studies on the correlation between CCECAI and hematological and immunological parameters raised a question about a robust conclusion regarding the diagnostic value of this correlation. Consequently, in the present statistical procedure, we conducted a meta-analysis of the correlation between CCECAI and CRP, NLR, and PLR in dogs with CE.

In the present study, the correlation between the CCECAI and NLR emerged as the strongest biomarker, with a pooled correlation of 0.41 and Fisher’s Z estimate of 0.436. This finding confirms the results obtained in dogs with chronic enteropathy [26,36,42]. Similarly, this finding is in accordance with prior meta-analyses showing NLR as a robust predictor of disease severity and survival across inflammatory and oncologic conditions in humans [43,44,45,46].

PLR showed a moderate association (pooled correlation 0.38), which was slightly weaker than NLR, but was still significant. Studies on autoimmune and inflammatory diseases in humans suggest that PLR has diagnostic and prognostic utility, although it is often less consistent than the NLR [47]. Several meta-analyses have confirmed the diagnostic and prognostic value of PLR in different disease conditions in humans [48,49,50]. The combined evaluation of NLR and PLR in dogs with inflammatory and oncologic conditions has also confirmed their diagnostic value [26,51]. In dogs with chronic enteropathy, the NLR and PLR increase, mainly due to systemic inflammation, immune dysregulation, and disease severity. A higher NLR and PLR have been reported in protein-losing and immunosuppressant-responsive enteropathies, and both ratios tend to normalize with effective therapy [26,36,42].

The present meta-analysis provides evidence of a consistent, small-to-moderate positive correlation between CRP and CCECAI, supporting the role of CRP as a biomarker of systemic inflammation. Although the strength of the association was modest (pooled r = 0.229), the statistical robustness across multiple independent studies highlights its clinical relevance. Interestingly, most of the included studies demonstrated significant correlations, with only one reporting a borderline non-significant result, suggesting that variability may be attributable to differences in the study design, sample size, or population characteristics. The absence of substantial publication bias further reinforces confidence in the pooled estimate. From a clinical perspective, these findings support the utility of the correlation between CRP and CCECAI as a supplementary diagnostic indicator, particularly when interpreted along with other inflammatory markers. Methodologically, Fisher’s Z transformation confirmed the stability of the pooled effect, underscoring the reliability of the results. Nevertheless, the moderate effect size indicates that CRP alone should not be considered a definitive predictor but rather a part of a broader panel of biomarkers. Similar studies have confirmed the diagnostic and prognostic value of CRP in dogs with different disease conditions [39,52,53]. In addition, a meta-analysis of the diagnostic value of CRP supports the findings of our study [54].

NLR showed substantial heterogeneity (I^2^ = 68%), reflecting the variability in study populations, disease contexts, and cut-off values. PLR demonstrated moderate heterogeneity (I^2^ = 41%), suggesting more consistency than NLR, but was still subject to contextual variation. However, CRP showed no heterogeneity (I^2^ = 0%), underscoring its stability across diverse clinical settings. This robustness makes CRP a reliable adjunct marker, even if its effect size is small. Heterogeneity is an inherent feature of meta-analyses, reflecting the diversity of clinical, methodological, and statistical characteristics across the included studies [55,56,57]. In the present meta-analysis, we propose that the heterogeneity could be attributed to differences in the sample size, methodology, and conditions of the study. Cochran’s Q statistics provide the degree of heterogeneity between studies. This helps to determine whether differences in the results of a study are due to chance or other factors. There is likely to be some heterogeneity in the research if the Q value is considerably greater than the degrees of freedom [56]. If the Q-statistic produces no dispersion in the effect size, I^2^ and tau-squared can provide alternative interpretations [57]. The heterogeneity test also defines the difference between studies attributed to sample errors [58,59,60,61].

Regarding publication bias, CRP showed evidence of publication bias. This finding was confirmed by the asymmetry of the funnel plot and the results of Egger’s test. However, the trim-and-fill analysis indicated only a modest reduction in effect size (0.22 to 0.19), suggesting that the overall conclusions remain acceptable. NLR did not show significant bias, and fail-safe N analysis suggested strong robustness (41 null studies were required to nullify significance). PLR presented mixed evidence: Egger’s test indicated asymmetry, while fail-safe N and Orwin’s tests supported stability. This implies that PLR’s effect may be somewhat inflated by small study effects but is still clinically relevant. Publication bias was not the only cause of funnel plot asymmetry. Other factors included true heterogeneity (small studies conducted in different contexts), poor methodological quality in smaller studies, and chance. Egger’s test has low power with few studies (<10); therefore, the results should be interpreted cautiously [62]. Publication bias often occurs in studies with relatively small sample sizes [63]. Since bias plays a significant role in the results of systematic meta-analyses, the detection of bias is important [64]. A funnel plot was created to show evidence of publishing bias. In this figure, the magnitude of the effect is usually plotted against the precision or standard errors [65]. A zero level of the regression slope from statistical meta-analysis only indicates no publication bias [66]. A strong correlation in the Begg test implies the presence of publication bias [67].

This study has some limitations. First, in this meta-analysis, there was a relatively small number of studies included. However, we followed the standard steps of analysis when there were few included studies [68]. Several meta-analytical studies in humans and veterinary medicine have included a few studies and provided reliable conclusions [25,35,69,70,71,72]. Second, a correlation was observed between CCECAI and CRP, NLR, and PLR only. Unfortunately, several variables were tested in correlation with CCECAI, but the number of studies on such variables was not more than two.

## 5. Conclusions

The results of the present meta-analysis indicate that the correlation between CCECAI and each of NLR and PLR may provide rapid and reliable diagnostic information about CIE in dogs. Future research should focus on the assessment of additional highly sensitive diagnostic markers and their correlation with CCECAI in dogs with CIE. Moreover, correlation of CCECAI with biomarkers in different types of CIE in dogs may be of clinical significance for the diagnosis of such disease in dogs.

## Figures and Tables

**Table 1:** Descriptive data of studies on the correlation between CCECAI and the CRP in dogs with chronic enteropathy.

Study	Sample Size	Study Design and Source	Correlation Coefficient	*p*-Value
Heilmann et al. [6]	127	Prospective case–control study on 127 dogs between August 2009 and July 2015 at the Veterinary Teaching Hospitals at TAMU or Purdue University, or at 1 of several other referral hospitals across the United States.	0.42	0.006
Benvenuti et al. [7]	165	Prospective multicenter study at two university veterinary hospitals (Pisa and Bologna universities) and at an Italian private veterinary clinic between January 2017 and September 2019.	-	0.06
Cagnasso et al. [8]	53	Retrospective study on 53 dogs with protein loss enteropathy between January 2019 and January 2022.	0.2463005	0.007
Gianella et al. [22]	91	Prospective case–control study on 91 dogs (51 diseased and 40 healthy) at Veterinary Teaching Hospital, Department of Veterinary Sciences, University of Turin, Italy from January 2021 to March 2022	0.28	0.042
Sattasathuchana et al. [35]	68	Prospective cross-sectional on 68 dogs with chronic inflammatory enteropathy at multiple veterinary clinics during 2012 and 2014 in USA.	0.4479	0.01

**Table 2:** Descriptive data of studies on the correlation between CCECAI and NLR in dogs with chronic enteropathy.

Study	Sample Size	Study Design and Source	Correlation Coefficient	*p*-Value
Benvenuti et al. [7]	41	Retrospective study on 41 dogs presented to University of Pisa, Italy, from January 2017 to January 2018	0.52	0.004
Becher et al. [27]	93	Prospective study on 93 dogs at two different veterinary centers: the Gastrointestinal Laboratory at the Small Animal Veterinary Teaching Hospital at Texas A&M University (TAMU, College Station, TX, USA; 2008–2015, cases from the TAMU Small Animal Veterinary Teaching Hospital or other tertiary veterinary centers across the United States) and the Department for Small Animals at the University of Leipzig (UL, Germany; 2013–2018).	0.41	0.0008
Cristóbal et al. [36]	16	A case–control study on 16 dogs diagnosed with Chronic inflammatory enteropathy at the Internal Medicine Unit of the Veterinary Teaching Hospital of the UEx (VTH-UEx).	0.52	0.0001
Cagnasso et al. [8]	53	Retrospective study on 53 dogs with protein loss enteropathy between January 2019 and January 2022	-	0.05

**Table 3:** Descriptive data of studies on the correlation between CCECAI and PLR in dogs with chronic enteropathy.

Study	Sample Size	Study Design and Source	Correlation Coefficient	*p*-Value
Cristóbal et al. [36]	16	A case–control study on 16 dogs diagnosed with Chronic inflammatory enteropathy at the Internal Medicine Unit of the Veterinary Teaching Hospital of the UEx (VTH-UEx).	0.53	0.0001
Mehain et al. [37]	44	A retrospective study on 22 dogs with chronic enteropathies at Washington State University Veterinary Teaching Hospital between September 2010 and November 2017	0.67	0.024
Pierini et al. [26]	41	Retrospective study on 41 dogs at multicentric study over a one-year study period	0.42	0.005
Pierini et al. [38]	41	Retrospective study on 41 immunosuppressant-responsive enteropathies from January 2018 and January 2019	0.033	0.036

**Table 4:** Results of meta-analysis on the correlation between CCECAI and CRP, NLR and PLR in dogs with chronic enteropathy.

Variable	CRP			NLR			PLR		
	Random Effect	*p*-Value	CI at 95%	Random Effect	*p*-Value	CI at 95%	Random Effect	*p*-Value	CI at 95%
Correlation (pooled effect size)	0.229	<0.001	0.14–0.31	0.41	<0.001	0.28–0.52	0.38	<0.001	0.23–0.50
Fisher’s (pooled effect size)	0.223	<0.001	0.14–0.32	0.436	<0.001	0.29–0.57	0.40	<0.001	0.24–0.56
Heterogenicity									
Q-statistics	2.86	0.58		9.44	0.024		5.07	0.166	
I^2^	0.00	0.58		68.24	0.024		26.8	0.17	
Z-value	5.14	<0.001		6.02	<0.001		4.95	<0.001	
Publication bias									
Begg and Mazumdar rank correlation	0.7	0.08		0.5	0.3		0.66	0.17	
Egger’s regression intercept	3.35	0.028	0.67–6.0	4.45	0.11	−2.76–1.67	4.35	0.030	0.98–7.73
Orwin’s fail-safe N	0.22			0.41			0.40		
Duval and Tweedie’s trim and fill	0.19		0.11–0.27	0.36		0.24–0.47	0.34		0.19–0.50

CCECAI: Canine Chronic Enteropathy Clinical Activity Index; CRP: C-reactive protein; NLR: neutrophil to lymphocyte ratio; PLR: platelet to lymphocyte ratio.

## Data Availability

The original contributions of this study are included in this article. Further inquiries can be directed to the corresponding authors.
